# The Role of Lipid Droplets in *Mortierella alpina* Aging Revealed by Integrative Subcellular and Whole-Cell Proteome Analysis

**DOI:** 10.1038/srep43896

**Published:** 2017-03-07

**Authors:** Yadong Yu, Tao Li, Na Wu, Ling Jiang, Xiaojun Ji, He Huang

**Affiliations:** 1 Jiangsu National Synergetic Innovation Center for Advanced Materials (SICAM), Nanjing Tech University, Nanjing, 211800, China; 2 College of Biotechnology and Pharmaceutical Engineering, Nanjing Tech University, Nanjing, 211800, China; 3 College of Food Science and Light Industry, Nanjing Tech University, Nanjing, 211800, China; 4 School of Pharmaceutical Sciences, Nanjing Tech University, Nanjing, 211800, China; 5 State Key Laboratory of Materials-Oriented Chemical Engineering, Nanjing Tech University, Nanjing, 211800, China

## Abstract

Lipid droplets (LDs) participate in many cellular processes in oleaginous microorganisms. However, the exact function of LDs in the *Mortierella alpina* aging process remains elusive. Herein, subcellular proteomics was employed to unveil the composition and dynamics of the LD proteome in the aging *M. alpina* for the first time. More than 400 proteins were detected in LDs and 62 of them changed expression significantly during aging. By combining the LD proteomic data with whole-cell data, we found that the carbohydrate metabolism and de novo lipid biosynthesis were all inhibited during aging of *M. alpina* mycelia. The up-regulation of fructose metabolism-related enzymes in LDs might imply that LDs facilitated the fructose metabolism, which in turn might cause pyruvate to accumulate and enter malate-pyruvate cycle, and ultimately, provide additional NADPH for the synthesis of arachidonic acid (ARA). Lysophospholipase and lecithinase were up-regulated in LDs during the aging process, suggesting that the phospholipids and lecithin were starting to be hydrolyzed, in order to release fatty acids for the cells. The impairment of the anti-oxidant system might lead to the accumulation of ROS and consequently cause the up-regulation of autophagy-related proteins in LDs, which further induces the *M. alpina* mycelia to activate the autophagy process.

Lipid droplets (LDs), are intracellular organelles characterized by large deposits of neutral lipids, and consist of a neutral lipid core surrounded by a phospholipid monolayer^1^,^2^,^3^. It has been shown that LDs serve as the energy reservoir of cells, and thus play a function in cellular adaptation during nutrient deprivation via mobilization and degradation of lipids. They also interact with other cellular processes, including protein storage, autophagy, lipid transport and metabolism^4^,^5^,^6^,^7^. An increasing body of evidence demonstrates that these functions are performed by LDs-associated proteins which are embedded in the phospholipid monolayer of LDs. Thus, efforts have been made to uncover the LD proteome in oleaginous microorganisms, as well as other species, and intriguing findings have been reported^5^,^7^,^8^,^9^. However, these LDs-related studies in oleaginous microorganisms usually only focused on analyzing the composition or changes of the LD proteome, whereas the association between the changes of the LDs proteome and broader cellular signal pathways was usually neglected. This lack of data hampers a detailed understanding of how the LD proteome changes during microbial responses to environmental or fermentation conditions, since the LD proteome is intimately linked to other organelles and cellular signal pathways^2^,^5^,^10^.

Arachidonic acid (ω-6,5,8,11,14-cis-eicosatetraenoic acid; ARA) is an important polyunsaturated fatty acid which shows various physiological functions in the human body and has broad applications in medicine, cosmetics, and other fields^11^,^12^,^13^. *Mortierella alpina*, a filamentous fungus, is viewed as a particularly good producer of ARA-rich oil^12^. The aging technology for *M. alpina*, which employ an additional culture step after regular fermentation, which is conducted in an environment without carbon source, has long been known as an effective way to improve ARA content^12^. Various aging methods have been elaborated, whereas attempts to find out the mechanism of ARA accumulation during aging, let alone to identify the dynamics and the precise roles of LD-associated proteins, are still limited.

Since LD-associated proteins are involved in various cellular processes, such as lipid metabolism and LD size control^4^,^14^, investigating the *M. alpina* LD proteins and their associations with the whole-cell signal pathways may be a key step to understand the exact mechanism of ARA accumulation in the aging *M. alpina* mycelia. Therefore, we used a subcellular proteomics approach to characterize the composition and changes of the LD proteome during the *M. alpina* aging process. Moreover, we further combined whole-cell proteome data published separately, with the LD proteome data from this study to identify the associations between the whole-cell signal pathways and the dynamics of the LD proteome in aging *M. alpina* cells. Our findings show that LD-associated proteins have diverse functions involved in lipid metabolism and the dynamics of LD proteome are influenced by some crucial signal pathways. These results will pave the way for a detailed understanding of the molecular mechanisms of *M. alpina* aging and help to improve the aging technology for ARA production.

## Results and Discussion

### Changes of mycelial morphology and ultrastructure during the aging process

**Figure 1:**
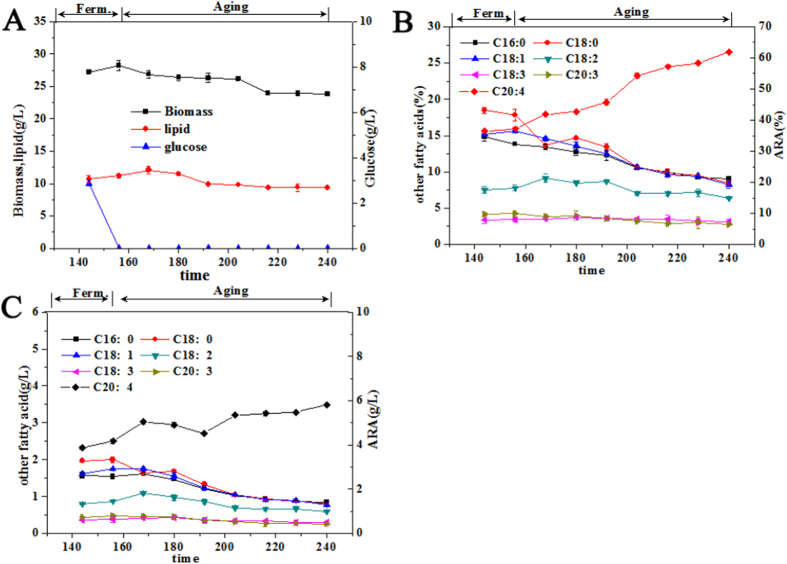
(**A**) Changes of cellular biomass, lipid concentration, and residual glucose; (**B**) Changes of the percentage of arachidonic acid (ARA) and other fatty acids in total fatty acids; (**C**) Changes of the concentrations of ARA and other fatty acids. C20:4 denotes ARA. (Reprinted with permission from (Yu, Y. *et al*. Mechanism of Arachidonic Acid Accumulation During Aging in Mortierella alpina: A Large-Scale Label-Free Comparative Proteomics Study. *J. Agric. Food Chem.* **64**, 9124–9134 (2016)). Copyright (2016) American Chemical Society).

In our previous work, we found that, *M. alpina* mycelia entered the aging period after around 156 h of fermentation, when glucose was exhausted (Fig. 1A). The ARA percentage increased from 37.2% to 62.0% and ARA concentration increased from 3.9 g/L to 5.8 g/L. Interestingly, both the percentages and the concentrations of the other fatty acids decreased (Fig. 1B,C)^15^.

**Figure 2:**
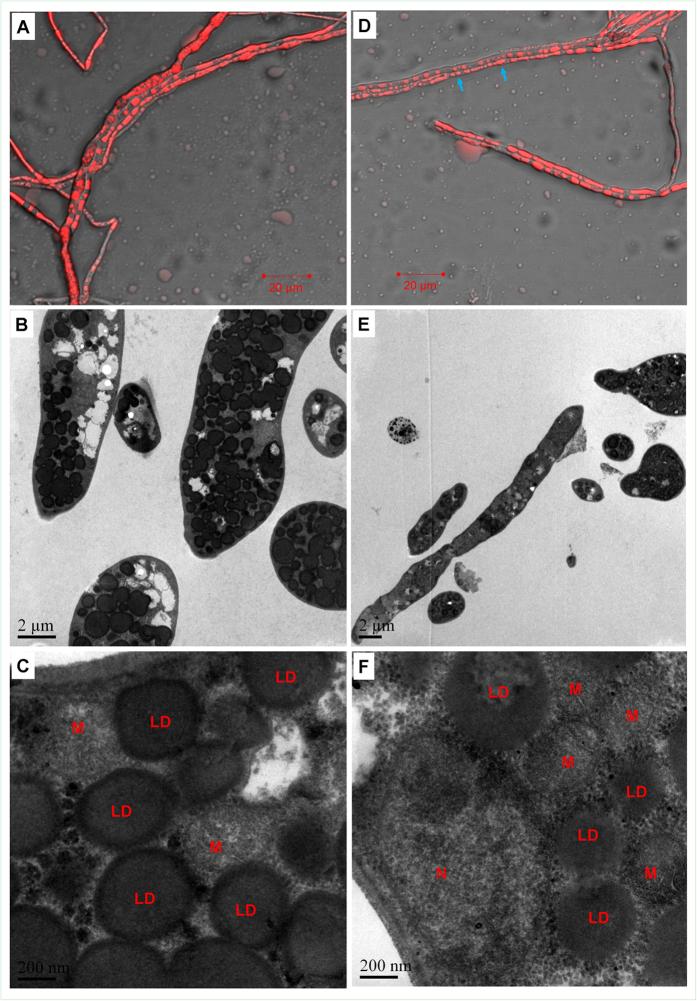
Fluorescence microscopic and TEM images of the mycelia harvested at the end of a regular fermentation process (156 h) (**A**–**C**) and the middle stage of the aging process (192 h) (**D–F**). N, M and LD denoted the nucleus, mitochondria and lipid droplets, respectively.

Herein, we further explored the changes of mycelial morphology and ultrastructure since they are intimately associated with the physiological status of microbial cells. At 156 h (the end-point of a regular fermentation process), most of the *M. alpina* mycelia displayed a normal, unbroken filamentous appearance (Fig. 2A). After staining with a lipid droplet specific probe (Nile Red), a large amount of red spheres were found in the mycelia (Fig. 2A). Lipid droplets (LDs) occupied a large proportion of the cell volume and a subset of them seemed to be fusing or budding (Fig. 2B,C). At 192 h (the middle stage of the aging process), most of the mycelia still seemed to be unbroken. However, some LDs in the mycelia disappeared or became smaller (indicated with blue arrows in Fig. 2D). TEM observation further confirmed that some LDs were shrunken. Interestingly, mitochondria seemed to aggregate around these shrunken LDs (Fig. 2F). Tarnopolsky *et al*. had found that mitochondria would relocate towards and interact with LDs more actively when the energy supply or fatty acid contents in the cells was insufficient^16^. It is well-known that when the energy supply for cells is insufficient, triacylglycerols (TAGs) stored in the LDs can be hydrolyzed to release fatty acids which will then be utilized by mitochondria to produce more ATP^6^. Taken together, we hypothesized that, when mycelia had no external carbon source and experienced a shortage of energy supply, mitochondria would relocate to the immediate proximity of the LDs, which would facilitate the mitochondrial utilization of the fatty acids stored in TAGs and ultimately, lead to the shrinkage of LDs.

### Characterization of lipid droplets

**Figure 3:**
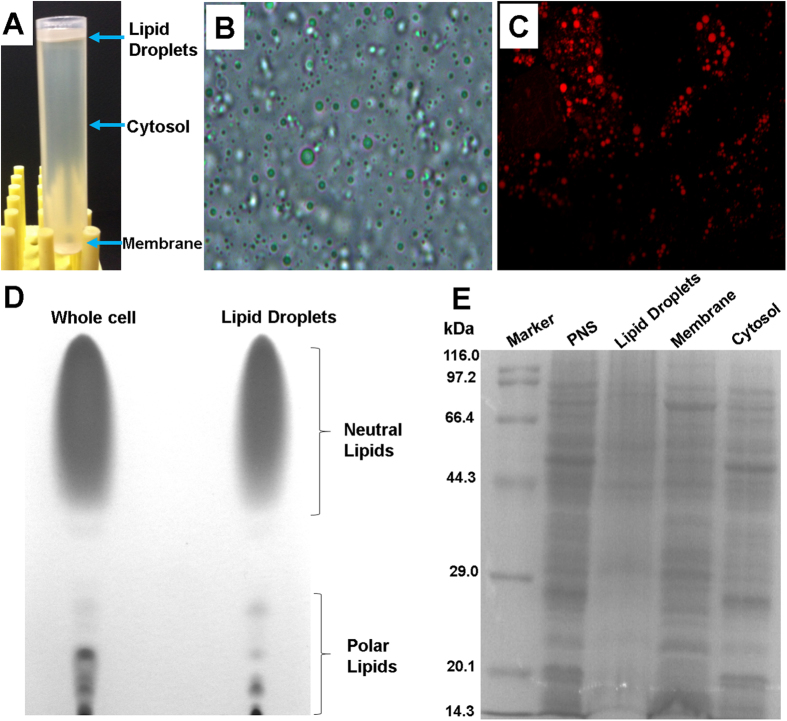
(**A**) Naked-eye appearance of a centrifuged post-nuclear supernatant (PNS) sample; (**B**) Light microscopy image of the isolated LDs; (**C**) Fluorescent microscope image of the isolated LDs; (**D**) Thin-layer chromatography of lipid samples from whole cells and LDs; (**E**) SDS-PAGE analysis of proteins from the PNS, LD, membrane, and cytosol fractions.

According to Ding and coworkers’ method^17^, we successfully isolated LDs in large quantity as the fraction on the top of a density gradient (Fig. 3A). The isolated LDs appeared to be spherical in structure (Fig. 3B). After staining with the lipid-specific probe Nile Red, the isolated LDs presented as red beads (Fig. 3C), which was consistent with the literatures^17^,^18^. Moreover, we found that the lipid samples from the isolated LDs had a significantly higher proportion of neutral lipids and a corresponding smaller proportion of polar lipids than the total lipid samples from the whole cells, which further confirmed the satisfactory purity of the isolated LDs (Fig. 3D). The SDS-PAGE results indicated that the protein profile of the LDs was unique and different from the other cellular fractions, including total membrane, cytosol, and post-nuclear supernatant (PNS), which further demonstrated that the isolated LDs were purified to a considerable degree (Fig. 3E).

### Label-free quantification of the lipid droplet proteome

Since the percentage and concentration of ARA increased remarkably at the middle stage of the aging process (192 h) (Fig. 1B,C), significant cellular events must have taken place in the *M. alpina* mycelia at this time point. Moreover, in our other work, we found that at the end of the aging process (240 h) the mycelia began to decompose and cytoplasm seemed to have leaked out^15^. We thus analyzed the LD proteome of mycelia at the middle stage of the aging process (192 h, aging group) and compared it to those at the end of regular fermentation (156 h, control group).

**Figure 4:**
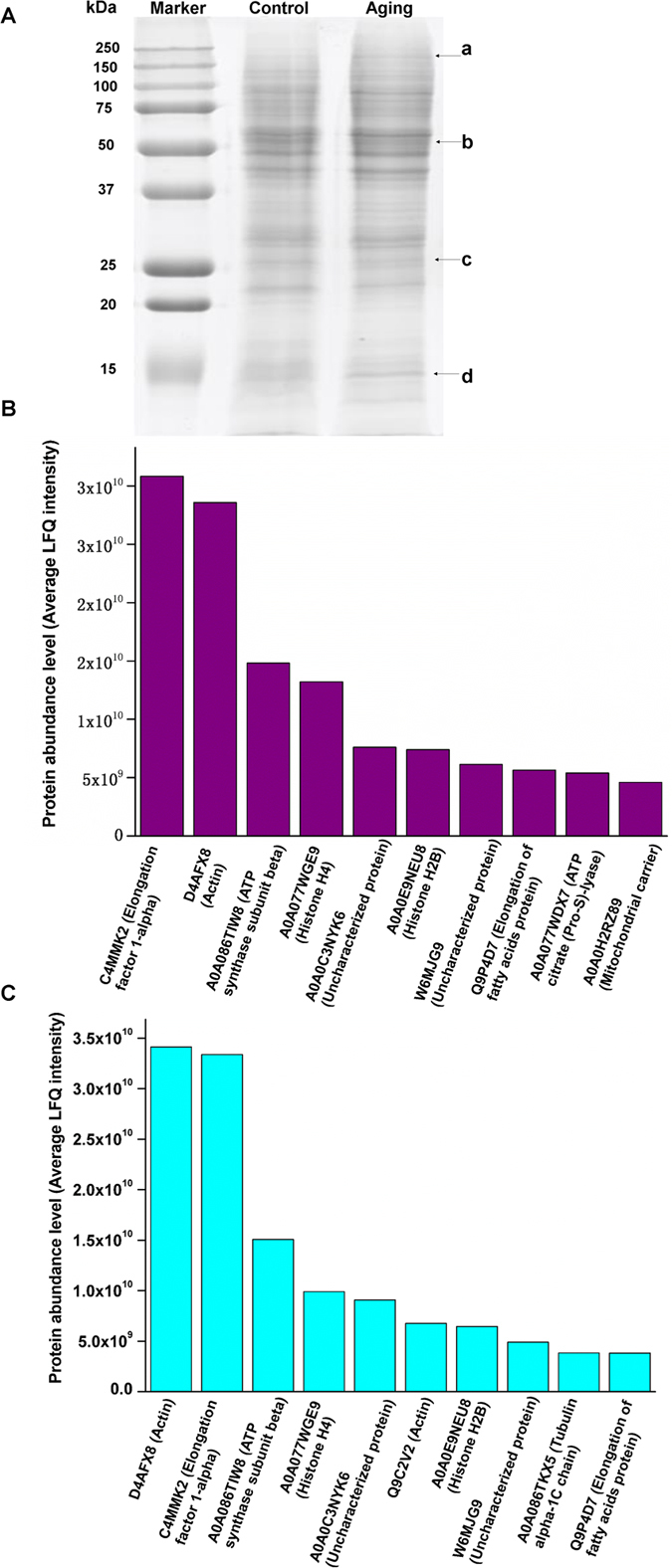
(**A**) SDS-PAGE analysis of the lipid droplet-associated proteins in the control and aging group; (**B–C**) Top 10 abundant lipid droplet-associated proteins in the control (**B**) and aging group (**C**).

**Figure 5:**
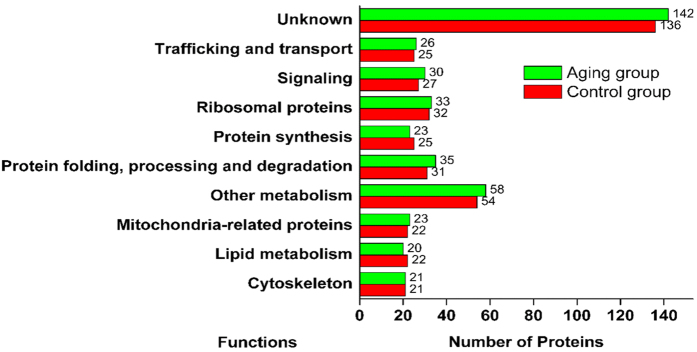
Functional catalogues of the lipid droplet-associated proteins.

After analyzing the LD-associated proteins via SDS-PAGE, we found that the electrophoretic bands of the control group were slightly different from those of the aging group (e.g., band a, b, c, d, Fig. 4A), suggesting that the LD proteome might have changed during aging. Using a large-scale label-free proteomics method, 395 and 411 proteins were detected in the LD proteomes from the control and aging group, respectively. The top 10 most abundant proteins in the *M. alpina* LD proteome were actin, elongation factor 1-alpha, ATP synthase subunit beta, Histone H4, Histone H2B, Tubulin alpha-1C chain and proteins related to the elongation of fatty acids (Fig. 4B,C). As shown in Fig. 5, the LD-associated proteins were further classified into ten groups by their function or localization. A large proportion of the LD associated proteins was still unknown due to as of yet insufficient efforts to investigate the proteins of filamentous fungi. Trafficking and signal-associated proteins were also identified, suggesting that LDs are key organelles for signal transduction, as well as lipid or protein trafficking and transport. Apart from our work, there are also reports in the literature that LDs contain proteins which facilitate the LDs participation in vesicle or membrane trafficking and inter-organellar communications^19^,^20^. Small Rab-type GTPases are the major class of this type of protein^21^. Furthermore, these proteins further make the LDs into hubs of fatty acid trafficking. For example, during autophagy, the released fatty acids can be shuttled through LDs as a way station before entering into mitochondria for ATP production^22^. Ribosomal proteins were also detected in the LD proteomes of *M. alpina* and other species^7^, suggesting that LDs may be a place of direct protein synthesis. This hypothesis was further supported by the fact that factors related to protein synthesis, protein folding and degradation were also found in the LD proteome. Moreover, the enrichment of proteins involved in lipid metabolism and other metabolic process (19% in the aging group and 19.2% in the control group) further proved that LDs in *M. alpina* play important roles in cellular metabolism, which is consistent with observations on other eukaryotic LDs^7^,^9^. Additionally, 23 and 22 mitochondria-related proteins were also identified in the aging and control groups, respectively, which might be due to contamination that may have resulted from structural or functional interactions between LDs and mitochondria^23^.

### Quantitative changes induced in the lipid droplet proteome during the aging process of *M. alpina*

**Figure 6:**
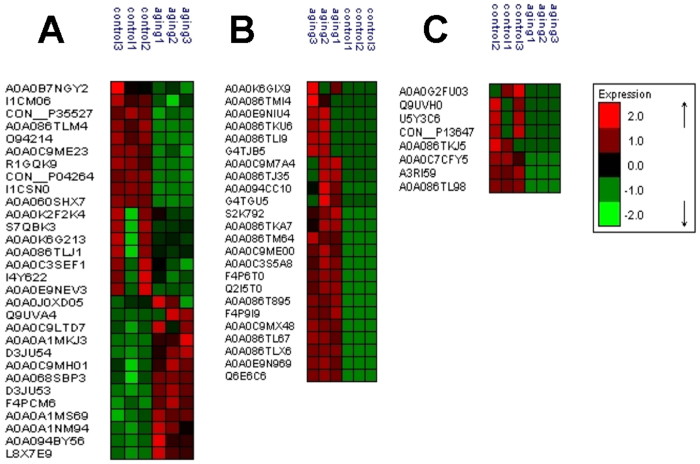
(**A**) cluster analysis of the significantly up- or down-regulated proteins in the aging group; (**B**) cluster analysis of the proteins newly arising during the aging process; (**C**) cluster analysis of the proteins which were not undetected in the samples from the aging process.

Based on the large-scale label-free comparative proteomics method and statistical filtration (fold change >2, *p* < 0.05), 30 proteins showed significant differences in abundance (13 up-regulated and 17 down-regulated) in the aging group as compared to the control group (Fig. 6A). Moreover, 24 newly-arising proteins were identified (Fig. 6B), and eight proteins became undetectable in the aging group (Fig. 6C). Overall, these results indicated that the expression profiles of the *M. alpina* LD proteome changed significantly during the aging process.

### Integrative analysis of the lipid droplet and whole-cell proteomes

The metabolic pathways containing enzymes expressed differentially in the LD proteome, were further analyzed using Blast2go software and the KEGG database. As shown in Table 1, these enzymes are part of various pathways, such as carbohydrate, as well as fatty, and amino acid metabolism, suggesting that the LDs of *M. alpina*, actively participate in metabolic regulation, as is true for other eukaryotes as well.

Different lines of evidence corroborate the idea that the LDs are intimately linked to other organelles and regulated by many intracellular signal pathways^2^,^5^,^10^. In the past decades, whole-cell proteome analysis has shown its outstanding potential in exploring whole-cell events, because proteins most often serve as the executors of specific physiological functions. Thus, a possible approach to fully understand the role of LDs and the association of changes of the LD proteome with many other cellular events might be a combined analysis of the LD proteome with the corresponding whole-cell proteome. With regard to this, an integrative analysis encompassing the LD and whole-cell proteomes of aging *M. alpina* mycelia was conducted to unveil the in-depth mechanism for how and why LD proteome changed during this process. The whole-cell proteome data were obtained from the same samples that were used in this study and have been described in detail in our another work^15^.

### Pathways related to carbohydrate metabolism

As shown in Table 1, the enzymes involved in most of the carbohydrate metabolism-related pathways, such as starch and sucrose metabolism, citrate cycle (TCA cycle), glycolysis and the pentose phosphate pathway, were down-regulated in both of the two proteome datasets. Since these pathways are responsible for supplying substrates and energy to biological processes, the inhibition of carbohydrate metabolism-related pathways suggested that the energy supply of the mycelia was insufficient in aging *M. alpina* cells. Since it has been found that mitochondria would relocate close to LDs when the cellular energy supply is experiencing a shortage^16^, the inhibition of these carbohydrate metabolic pathways might further explain why mitochondria relocated to the proximity of LDs in the aging *M. alpina* mycelia (Fig. 2F). In addition, as shown in Fig. 2F, the mitochondria in the mycelia were found to aggregate and fuse when the mycelia were undergoing carbon starvation during the aging process. Accumulating evidence demonstrates that, when the carbohydrate metabolism is weaken in starved cells, mitochondria are often highly fused to form an interwoven mitochondrial network, which seems to be a way to ensure that the fatty acids released from the LDs are evenly distributed throughout the mitochondria, consequently promoting balanced mitochondrial respiration^22^.

Interestingly, two enzymes related to the metabolism of fructose and mannose (EC:4.2.1.47–4,6-dehydratase and EC:1.1.1.271-synthase) were up-regulated in the LDs during the aging process (Table 1). Since a label-free quantitative proteomics method was utilized in this work, the abundance of the differentially expressed proteins could be quantified using their label-free quantification (LFQ) intensities^24^. Thus, to test the abundances of EC:4.2.1.47–4,6-dehydratase and EC:1.1.1.271-synthase, we examined the LFQ intensities of these two enzymes in our proteomics data. We found that the LFQ intensities of EC:4.2.1.47–4,6-dehydratase and EC:1.1.1.271-synthase in the aging group were 3.36-fold and 3.06-fold higher than those in the control group, respectively (Supplementary Figure S1). Therefore, this result further confirmed that these two enzymes were significantly up-regulated in the aging group, suggesting that LDs might facilitate fructose and mannose metabolism. Since most of the mannose in eukaryotes is catabolized via phosphomannose isomerase (MPI) to provide energy^25^, this result hinted that LDs might facilitate mannose metabolism which can provide additional energy for the aging *M. alpina* mycelia during carbon starvation.

In our previous work, we found that *M. alpina* mycelia could accumulate sucrose prior to entering the aging process and the accumulated sucrose was subsequently catabolized quickly at the early stage of aging^26^. The D-fructose-6-phosphate derived from the catabolism of sucrose can enter into the EMP pathway, which may further generate additional pyruvate and it has in fact been shown that high fructose consumption can lead to excess pyruvate production^27^. On the other hand, in the whole-cell proteome data, dihydrolipoyl dehydrogenase (EC:1.8.1.4-dehydrogenase) and phosphoenolpyruvate carboxykinase (EC:4.1.1.49-carboxykinase(ATP)), which are responsible for the conversion of pyruvate to phosphoenolpyruvate and acetyl-CoA^28^, were also down-regulated. Taken together, these results imply that additional pyruvate might be generated and at the same time, pyruvate conversion may be suppressed, so that pyruvate would be accumulated in the cells. Our previous finding had demonstrated that pyruvate concentrations indeed increased during the aging process of *M. alpina*^26^. The accumulated pyruvate might enter the malate-pyruvate cycle, which provides NADPH for fatty acid dehydrogenation as is needed to synthesize ARA^29^. This might be an explanation for the phenomenon of increased ARA content in aging *M. alpina*.

### Pathways related to fatty acid metabolism

As shown in Table 1, fatty-acid synthase (EC:2.3.1.85-synthase) and acetyl-CoA-carboxylase (EC:6.4.1.2-carboxylase), which are involved in *de novo* fatty acid biosynthesis, were found to be down-regulated both in the LD and the whole-cell proteomes, suggesting that *de novo* fatty acid biosynthesis was significantly weakened in *M. alpina* during the aging period. This was also confirmed by the results of lipid analysis (Fig. 1). In agreement with the result reported by Hao^30^, acyl-CoA desaturase (EC:1.14.19.1-desaturase), delta 6 fatty acid desaturase (EC:1.14.19.3-desaturase) and delta 12 desaturase (EC:1.14.19.6 -desaturase), the key enzymes for the biosynthesis of unsaturated fatty acids, were all down-regulated in the LD and whole-cell proteomes from aging cells. Interestingly, the ARA content was still increased in spite of the decrease of these fatty acid desaturases. This result might be due to the fact that, even though these fatty acid desaturases were down-regulated, their initial contents were already high and thereby, ARA could still be synthesize due to their lingering enzyme activity^15^. Moreover, EC:4.2.1.17-hydratase, an enzyme involved in the biosynthesis of unsaturated fatty acids, fatty acid elongation and alpha-linolenic acid metabolism, was found to be remarkably increased in abundance, suggesting that this enzyme might be a key player in the elongation and dehydrogenation of unsaturated fatty acids during the aging process.

Notably, lysophospholipase (EC:3.1.1.5-lysophospholipase) was up-regulated in the LD proteome of the aging cells, while it remained undetectable in the whole-cell proteome (Table 1). Lysophospholipase, also called phospholipase B, which can release fatty acids from phospholipids, is considered to be a vital participant in phospholipid turnover^31^,^32^. Moreover, lecithinase (EC:3.1.1.5-lecithinase B), which is involved in glycerophospholipid metabolism and hydrolyzes lecithin to release fatty acids^33^, was also up-regulated in the LDs. Therefore, we hypothesized that lysophospholipase and lecithinase located in the LDs could catalyze the conversion of phospholipids or lecithin during the *M. alpina* aging process, so that the fatty acids could be released and utilized by mitochondria via beta-oxidation to provide energy for the cells to adapt to these adverse conditions.

### Amino acid and other metabolic pathways

As shown in Table 1, in the whole-cell proteome data, many enzymes related to amino acid degradation (e.g., EC:4.2.1.17-hydratase, EC:1.1.1.35 -dehydrogenase, EC:1.1.1.35-dehydrogenase), as opposed to those related to amino acid biosynthesis enzymes (e.g., EC:3.5.3.1-arginine amidinase, EC:4.2.1.33-dehydratase), were up-regulated. It has been demonstrated that a certain amount of amino acids is released via protein degradation and used for respiration when carbohydrate metabolism is inhibited^34^. These results suggested that increased amounts of proteins would be degraded during the aging process and amino acid degradation might thus be an important compensatory mechanism in cells undergoing carbon starvation. The number of differentially expressed enzymes related to the amino acid metabolism was however much smaller in the LD than in the whole-cell proteome dataset (Table 1), suggesting that LDs did not exert significant regulation on the metabolism of amino acids.

In addition, some differentially expressed enzymes related to other metabolic pathways (e.g., amino sugar and nucleotide sugar metabolism, purine metabolism) were also found in both the LD and the whole-cell proteome datasets. Notably, most of the enzymes involved in glutathione metabolism and oxidative phosphorylation were significantly down-regulated. Glutathione plays a key role in preventing damage caused by reactive oxygen species (ROS) in fungi^35^. Therefore, the down-regulation of the enzymes related to glutathione metabolism implied that the anti-oxidant system may be malfunctioning and ROS contents might increase simply due to a lack of ROS scavengers. In our another work, we confirmed that the ROS contents in the *M. alpina* mycelia remarkably increased during the aging process^15^. On the other hand, we further found that an autophagy-related protein (protein ID: A0A0E9N969) was newly expressed in the LDs during the aging process (Fig. 6B). Since accumulating evidence points to the essential role of ROS in the activation of autophagy^36^, we hypothesized that during the aging process, the impairment of the anti-oxidant system led to the accumulation of ROS, which further induced the expression of autophagy-related proteins in the LDs and steered the *M. alpina* mycelia towards autophagy. We previously found that some of the *M. alpina* mycelia appeared to decompose at the end of the aging process^15^. Since autophagy is a tightly controlled degradation process in which eukaryotic cells will digest components of their own cytoplasm^37^, our previous finding further supported the notion that during the aging process, *M. alpina* mycelia were undergoing active, and perhaps even runaway autophagy. Overall, the expression of autophagy-related proteins in LDs suggests that these organelles may themselves participate in the autophagy mechanism of *M. alpina*, and thus possess a nutrient recycling role necessary to prolong the survival of mycelia undergoing carbon starvation^38^.

## Conclusions

In summary, we found that the morphology of LDs in *M. alpina* mycelia significantly changed during the aging process. Using the subcellular proteomics method, the composition and dynamics of the LD proteome in aging *M. alpina* cells was investigated. *M. alpina* LDs contain a large number of different enzymes which participate in diverse metabolic pathways. By combining the LD proteomic data with whole-cell proteomic data, we found that enzymes related to carbohydrate metabolism and de novo lipid biosynthesis were all suppressed. The upregulation of enzymes related to fructose metabolism implies that LDs facilitate fructose utilization, which might cause pyruvate to accumulate, enter the malate-pyruvate cycle, and ultimately, provide additional NADPH for the biosynthesis of ARA. The increased expression of lysophospholipase and lecithinase suggests that the LDs started to hydrolyze their phospholipids and lecithin, releasing the resulting fatty acids for mitochondrial energy metabolism. At the same time, the impairment of antioxidant systems might lead to the accumulation of ROS, which in turn can induce the expression of autophagy-related proteins in the LDs, which might further mediate the activation of autophagy in *M. alpina* mycelia. We believe these findings will be helpful for deciphering the exact function of LDs during the *M. alpina* aging process and pave new ways for the efficient production of ARA and other polyunsaturated fatty acids via controlled mycelial aging.

## Methods

### Microorganism and media

*M. alpina* R807 (CCTCC M2012118) was purchased from the China Centre for Type Culture Collection. The potato dextrose agar consisted of (g/L): potato extract 200; glucose 25; agar 20. The seed medium contained (g/L): glucose 30; yeast extract 6; NaNO_3_ 3; KH_2_PO_4_ 3; MgSO_4_.7H_2_O 0.5. The fermentation medium contained (g/L): glucose 80, yeast extract 10, KH_2_PO_4_ 4, NaNO_3_ 3, MgSO_4_.7H_2_O 0.6.

### Culture and aging conditions

The culture and aging conditions for *M. alpina* were the same as those reported in our previous work^26^. Briefly, a small amount of *M. alpina* mycelia was taken from a seed tube containing 30% glycerol and stored at −80 °C, to transferred onto PDA slants. The cultures were subsequently incubated in an electro-thermal incubator for one week at 25 °C. When the PDA slant surface was covered with white mycelia, the mycelia were harvested for seed culture. 500 mL baffled flasks containing 100 mL fresh medium were used for seed and fermentation culture. Seed culture was conducted for 24 h, after which 10% (v/v) of the culture broth was used to inoculate the fermentation broth. After regular fermentation, mycelia were cultured continuously without carbon source to induce the aging process. All cultivations were conducted at 25 °C, initial pH 6.0, under constant orbital shaking at 125 rpm.

### Confocal and transmission electron microscopy observations

The experiments were conducted according to a reported method^39^, as follow: the harvested mycelia were resuspended in glycerol to yield a concentration of 0.1 g/mL, and an aliquot of 5 μL of Nile Red stock solution (0.4 mg/mL, J & K Scientific Ltd, China), was added to 3 mL of the mycelia-in-glycerol suspension and shaken gently for 1 min. After incubation in darkness for 5–10 min at room temperature, the samples were observed under a confocal microscope (FV1000, Olympus, Japan). The excitation wavelength was set at 488 nm and the emission wavelength scanning range was set between 500 and 750 nm.

The samples for transmission electron microscopy (TEM) observation were prepared as follows: after being cut into small pieces, mycelia were prefixed in 2.5% (w/v) glutaraldehyde in PBS (pH 7.4) at 4 °C. In order to remove residual glutaraldehyde, 0.1 M PBS (pH7.4) was used to wash the samples three times for 15 min each. Subsequently, 2% (w/v) osmium tetroxide (EM grade, Nakalai Tesque, Japan) was used to post-fix the samples at room temperature for 1 h. Sample dehydration was conducted via subsequent rinses with serially higher ethanol concentrations of 35%, 50%, 70%, 90%, 95% and 100%. After embedding in epoxy resin (Sigma, USA), samples were prepared as 90 nm sections using an EM UC6 Ultramicrotome (Leica, Germany). After double staining with uranyl acetate (Sigma, USA) and lead citrate (Sigma, USA), the sections were observed using a JEM-1011 TEM (JEOL, Japan).

### Isolation and characterization of lipid droplets

Lipid droplets (LDs) were isolated according to the method reported by Ding *et al*. with minor modifications^17^. Briefly, mycelia were collected from 100 mL of culture-broth, after which the mycelial pellets were washed three times in 35 mL of PBS and resuspended in 35 mL of Buffer A (20 mM tricine, 250 mM sucrose, pH 7.8) with 0.2 mM PMSF (phenylmethylsulfonyl fluoride, Sigma, USA). Samples were kept on ice for 20 min and homogenized four times at 4 °C using a Nano Homogenizer (AH100B, Ats Engineering, China) at 700 bar. The homogenate was centrifuged at 3000 g for 10 min at 4 °C to remove nuclei, cell debris and unbroken cells. The resulting post-nuclear supernatant (PNS) fraction was transferred into a 50 mL ultra-centrifuge tube. 8 mL of the PNS fraction was added into an SW40 tube and overlaid with 2 mL of buffer B (20 mM HEPES, 100 mM KCl, 2 mM MgCl_2_, pH7.4), after which the samples were centrifuged at 182000 g for 1 h at 4 °C. The floating LDs were collected in a 1 mL Eppendorf tube and retained on ice during the following washing steps. 1 mL of the solution was carefully taken from the middle of the gradient and further centrifuged at 270000 g for 30 min at 4 °C, and the resulting supernatant was collected as the cytosol fraction for determination of purity. The pellet at the bottom of the tube was washed three times with 1 mL of buffer B and resuspended in 1 mL of buffer B to obtain the total membrane fraction. The isolated LDs were suspended in buffer B and stained with Nile Red solution (J & K Scientific Ltd, China) or left unstained, for observation under an optical microscope (Leica, Germany) and a confocal microscope (FV1000, Olympus, Japan).

Neutral lipids and polar phospholipids were separated by silica gel thin-layer chromatography using a hexane-diethyl ether-acetic acid (80:20:1, vol:vol:vol) and a chloroform-methanol-acetic acid-H_2_O (75:13:9:3, vol:vol:vol:vol) solvent system, respectively. The samples were visualized using iodine vapor.

According to the reported method^17^, one milliliter of cell lysis buffer (50 mM Tris-HCl, 1.0 mM EDTA, 150 mM NaCl, 0.1% SDS) was added to the LD samples and the proteins extracted by ultrasonic disruption using an ultrasonicator (Ningbo Scientz Biotechnology Company, China) set to 20% duty cycle, 200 W power output, for 10 min, followed by centrifugation at 10000 g for 10 min. After denaturation for 5 min at 95 °C, the protein samples of the LD, membrane, cytosol, and PNS fractions were separated on a 10% polyacrylamide gel and stained with colloidal blue (Thermo Fisher Scientific, USA) using standard methods for comparing the protein profiles.

### Lipid droplet proteomics experiment

#### Isolation of lipid droplet-associated proteins

The protein samples of the LDs were separated by SDS-PAGE, the identified LD protein bands cut out of the gel, destained twice with 200 μL acetonitrile–25 mM ammonium bicarbonate (2:3, vol:vol) and washed 3 times with UA buffer (8 M Urea, 150 mM Tris-HCl pH 8.0) for protein digestion.

### Protein digestion

Protein digestion was conducted using the methods described by Wisniewski *et al*.^40^. In short, 200 μL UA buffer (8 M Urea, 150 mM Tris-HCl pH 8.0) was utilized to remove the low-molecular-weight components by repeated ultrafiltration. The samples were supplemented with 100 μL 0.05 M iodoacetamide (Sigma, USA) in UA buffer to block reduced cysteine residues and incubated for 20 min in the dark. The filter was washed three times with 100 μL UA buffer and twice with 100 μL 25 mM NH_4_HCO_3_. The resulting protein suspension was combined with 40 μL 25 mM NH_4_HCO_3_ containing 3 μg trypsin (Promega, USA) and incubated at 37 °C overnight for digestion. The resulting peptide concentration was determined via the optical density at 280 nm.

### Liquid chromatography-electrospray ionization tandem mass spectrometric analysis (LC-ESI-MS/MS)

C18 Cartridges (Sigma, USA) were used to desalt the peptides in each sample, which were subsequently concentrated by vacuum centrifugation and reconstituted in 40 μL 0.1% trifluoroacetic acid (Sigma, USA). To conduct the MS experiments on a Q Exactive mass spectrometer (Thermo Scientific, USA), samples comprising 5 μg of total peptides was loaded onto a the C18-reversed phase column (Thermo Scientific, USA) in buffer A (2% acetonitrile, 0.1% formic acid) and then separated with a linear gradient of buffer B (80% acetonitrile, 0.1% formic acid). To obtain the MS data, a data-dependent top10 method was used to choose the most abundant precursor ions from the dynamic survey scan for HCD fragmentation. The target value was determined based on predictive Automatic Gain Control. For each sample, the MS experiments were conducted three times.

### Whole-cell proteomics experiment

To obtain the whole-cell proteomics data, aliquots of the mycelia harvested by centrifugation for the lipid droplet proteomics experiment were used. The experimental protocol and whole-cell proteomics data are presented in other work^15^. Briefly, cell pellets were resuspended in 200 μL of lysis buffer (4% SDS, 100 mM DTT, 150 mM Tris-HCl pH 8.0) on ice. Cells were disrupted by agitation using a Fastprep-24^®^ homogenizer (MP Biomedical, USA), and subsequently boiled for 5 min. The samples were then ultrasonicated and boiled for another 5 min. After centrifugation at 14 000 rpm for 15 min, the resulting supernatants were collected and the corresponding protein concentrations were measured using a BCA Protein Assay Kit (Bio-Rad, USA) with BSA as standard. The protocols for protein digestion and LC-ESI-MS/MS were same as above.

### Bioinformatics analysis

#### Sequence database search and data analysis

The MaxQuant software suit (version 1.3.0.5) was used to analyze the MS data, which were searched against the UniProtKB database (uniprot_fungi incertae sedis_752077_20160118.fasta, 752077 total entries). An initial search was set at a precursor mass window of 6 ppm. This search was conducted with the enzymatic cleavage rule of Trypsin/P with a maximum of two missed cleavage sites allowed. Carbamidomethylation of cysteines was defined as fixed modification, while protein N-terminal acetylation and methionine oxidation were defined as variable modifications. The cutoff of the global false discovery rate (FDR) for peptide and protein identification was set at 0.01. Label-free quantification was conducted in MaxQuant according to the methods described previously^41^. The sequence data of the target proteins were bundled in batches which were retrieved from the UniProtKB database. The retrieved sequences were searched against the SwissProt database using the NCBI BLAST+ client software (version 2.2.28). The top 10 blast hits with E-values of less than 1*e*-3 were retrieved for each query sequence and finally loaded into Blast2GO (version 2.7.2) to analyze the related KEGG pathways^42^,^43^,^44^.

## Additional Information

**How to cite this article:** Yu, Y. *et al*. The Role of Lipid Droplets in *Mortierella alpina* Aging Revealed by Integrative Subcellular and Whole-Cell Proteome Analysis. *Sci. Rep.* **7**, 43896; doi: 10.1038/srep43896 (2017).

**Publisher's note:** Springer Nature remains neutral with regard to jurisdictional claims in published maps and institutional affiliations.

## Supplementary Material

Supplementary Information

## Figures and Tables

**Table 1:** Pathways encompassed by the differentially expressed enzymes in the LD proteome and the whole-cell proteome.

Pathway	Differentially expressed enzymes in LD proteome	Differentially expressed enzymes in whole-cell proteome
***Carbohydrate Metabolism Related Pathways***		
Starch and sucrose metabolism	EC:2.4.1.25-disproportionating enzyme ↓ EC:2.7.7.9-uridylyltransferase ↓ EC:3.2.1.33-amylo-1,6-glucosidase ↓	EC:3.2.1.28-trehalase ↓ EC:2.4.1.1-phosphorylase ↓ EC:5.4.2.2-(alpha-D-glucose-1,6-bisphosphate-dependent) ↓ EC:2.7.7.9-uridylyltransferase ↓
Galactose metabolism	EC:2.7.7.9-uridylyltransferase ↓ EC:2.7.7.64-uridylyltransferase ↓	EC:2.7.7.64-uridyltransferase ↓ EC:2.7.7.9-uridylyltransferase ↓ EC:5.4.2.2-(alpha-D-glucose-1,6-bisphosphate-dependent) ↓
Citrate cycle (TCA cycle)	EC:2.3.3.8-citrate synthase ↓	EC:1.8.1.4-dehydrogenase ↓ EC:4.1.1.49-carboxykinase(ATP) ↓ EC:1.2.4.2-dehydrogenase(succinyl-transferring) ↓ EC:1.1.1.42-dehydrogenase(NADP+) ↓ EC:1.1.1.41-dehydrogenase(NAD+) ↓
Pentose and glucuronate interconversions	EC:2.7.7.9-uridylyltransferase ↓ EC:2.7.7.64-uridylyltransferase ↓	EC:2.7.7.9-uridylyltransferase ↓ EC:2.7.7.64-uridylyltransferase ↓
Pyruvate metabolism		EC:1.8.1.4-dehydrogenase ↓ EC:6.4.1.2-carboxylase ↓ EC:4.1.1.49-carboxykinase(ATP) ↓ EC:1.1.1.39-dehydrogenase(decarboxylating) ↓ EC:1.1.1.38-dehydrogenase(oxaloacetate-decarbosylating) ↓
Glycolysis		EC:5.4.2.2-(alpha-D-glucose-1,6-bisphosphate-dependent) ↓ EC:4.2.1.11-hydratase ↓ EC:4.1.1.49-carboxykinase(ATP) ↓ EC:1.8.1.4-dehydrogenase ↓
Pentose phosphate pathway		EC:5.4.2.2-alpha-D-glucose-1,6-bisphosphate-dependent ↓
Fructose and mannose metabolism	EC:4.2.1.47–4,6-dehydratase ↑ EC:1.1.1.271-synthase ↑	
***Fatty Acid Metabolism Related Pathways***		
Fatty acid biosynthesis	EC:2.3.1.85-synthase ↓ EC:6.4.1.2-carboxylase ↓	EC:6.4.1.2-carboxylase ↓
Biosynthesis of unsaturated fatty acids	EC 1.14.19.1-desaturase ↓ EC 1.14.19.3-desaturase ↓ EC 1.14.19.6-desaturase ↓	EC 1.14.19.3-desaturase ↓ EC:4.2.1.17-hydratase ↑
Glycerophospholipid metabolism	EC:3.1.1.5-lecithinase B ↑	EC:2.7.8.8-O-phosphatidyltransferase ↓
Lipid catabolic process	EC 3.1.1.5-lysophospholipase ↑	
Fatty acid elongation		EC:4.2.1.17-hydratase ↑
Propanoate metabolism		EC:1.8.1.4-dehydrogenase ↓ EC:6.4.1.2-carboxylase ↓ EC:4.2.1.17-hydratase ↑
Alpha-linolenic acid metabolism		EC:4.2.1.17-hydratase ↑
Glyoxylate and dicarboxylate metabolism	EC:6.3.1.2-ligase ↑ EC:2.1.2.1-hydroxymethyltransferase ↑	
***Amino Acid Metabolism Related Pathways***		
Cysteine and methionine metabolism	EC:2.5.1.6-adenosyltransferase ↓	EC:3.3.1.1-S-adenosylhomocysteine synthase ↓
Phenylalanine, tyrosine and tryptophan biosynthesis	EC:2.5.1.54-synthase ↓	EC:4.2.1.20-synthase ↓
Arginine biosynthesis	EC:6.3.1.2-ligase ↑	EC:6.3.4.5-synthase ↑ EC:3.5.3.1-arginine amidinase ↓
Glycine, serine and threonine metabolism	EC:2.1.2.1-hydroxymethyltransferase ↑	EC:4.2.1.20-synthase ↓ EC:1.8.1.4-dehydrogenase ↓ EC:1.1.1.95-dehydrogenase ↓ EC:2.7.8.8-O-phosphatidyltransferase ↓
Alanine, aspartate and glutamate metabolism	EC:6.3.1.2-ligase ↑	EC:2.6.1.16-transaminase(isomerizing) ↓ EC:6.3.4.5-synthase ↑ EC:1.4.1.14-synthase ↓ EC:1.4.1.14-synthase(NADH) ↓ EC:1.4.1.13-synthase(NADPH) ↓
Cyano amino acid metabolism	EC:2.1.2.1-hydroxymethyltransferase ↑	
Valine, leucine and isoleucine degradation		EC:1.8.1.4-dehydrogenase ↓ EC:4.2.1.17-hydratase ↑ EC:1.1.1.35-dehydrogenase ↑ EC:1.1.1.35-dehydrogenase ↑
Lysine degradation		EC:1.2.4.2-dehydrogenase(succinyl-transferring) ↓ EC:4.2.1.17-hydratase ↑ EC:1.1.1.35-dehydrogenase ↑
Tryptophane metabolism		EC:1.2.4.2-dehydrogenase(succinyl-transferring) ↓ EC:4.2.1.17-hydratase ↑ EC:1.1.1.35-dehydrogenase ↑
Beta-alanine metabolism		EC:4.2.1.17-hydratase ↑
Arginine and proline metabolism		EC:3.5.3.1-arginine amidinase ↓
Valine, leucine and isoleucine Biosynthesis		EC:4.2.1.33-dehydratase ↓
Phenylalanine metabolism		EC:4.2.1.17-hydratase ↑
Histidine metabolism		EC:4.2.1.19-dehydratase ↓
***Other Metabolic Pathways***		
Amino sugar and nucleotide sugar metabolism	EC:2.7.7.9-uridylytransferase ↓ EC:2.7.7.64-uridylyltransferase ↓ EC:4.2.1.47–4,6-dehydratase ↑ EC:1.1.1.271-synthase ↑	EC:3.5.99.6-deaminase ↑ EC:2.6.1.16-transaminase(isomerizing) ↓ EC:2.7.7.64-uridylyltransferase ↓ EC:2.4.1.16-synthase ↑ EC:5.4.2.2-(alpha-D-glucose-1,6-bisphosphate-dependent) ↓ EC:2.7.7.9-uridylyltransferase ↓
Purine metabolism	EC:3.6.1.3-adenylpyrophosphatase ↑ EC:3.6.1.15-phosphatase ↑	
Pantothenate and CoA biosynthesis	EC:2.7.8.7-synthase ↓	
Glutathione metabolism		EC:1.1.1.42-dehydrogenase(NADP+) ↓ EC:1.11.1.15-thioredoxin peroxidase ↓
Oxidative phosphorylation		EC:1.6.99.3-dehydrogenase ↓ EC:1.6.5.3-reductase (H^+^-translocating) ↓ EC:3.6.1.1-diphosphatase ↑ EC:3.6.3.6-ATPase ↓

Note: Up-regulated enzymes were labelled with ↑; down-regulated enzymes were labelled with ↓.
