# Supplementary material for: The Role of Lipid Droplets in Mortierella alpina Aging Revealed by Integrative Subcellular and Whole-Cell Proteome Analysis
Source: Sci Rep. 2017 Mar 7;7:43896. doi: 10.1038/srep43896 (PMC5339828; doi:10.1038/srep43896)
Supplement: Supplementary Information [file srep43896-s1.pdf]

The Role of Lipid Droplets in *Mortierella alpina* Aging Revealed by Integrative Subcellular and Whole-Cell Proteome Analysis

Yadong Yu[1,#], Tao Li[2,#], Na Wu[2,#], Ling Jiang[3,*], Xiaojun Ji[1,2,*], He Huang[4,5,*]

[1] Jiangsu National Synergetic Innovation Center for Advanced Materials (SICAM), Nanjing Tech University, Nanjing, 211800, China

[2] College of Biotechnology and Pharmaceutical Engineering, Nanjing Tech University, Nanjing, 211800, China

[3] College of Food Science and Light Industry, Nanjing Tech University, Nanjing, 211800, China

[4] School of Pharmaceutical Sciences, Nanjing Tech University, Nanjing, 211800, China

[5] State Key Laboratory of Materials-Oriented Chemical Engineering, Nanjing Tech University, Nanjing, 211800, China

[#] These authors contributed equally to this work.

[*] Corresponding authors:

Ling Jiang, Ph.D

Associated Professor

College of Food Science and Light Industry, Nanjing Tech University

No.30 Puzhu South Road, Nanjing, 211800, China

Tel: (86) 25-58139942

Email: jiangling@njtech.edu.cn

Xiaojun Ji, Ph.D

Associated Professor

Jiangsu National Synergetic Innovation Center for Advanced Materials (SICAM),

Nanjing Tech University

College of Biotechnology and Pharmaceutical Engineering, Nanjing Tech University

No.30 Puzhu South Road, Nanjing, 211800, China

Tel: (86) 25-58139942

Email: xiaojunji@njtech.edu.cn

He Huang, Ph.D.

Professor

School of Pharmaceutical Sciences, Nanjing Tech University

State Key Laboratory of Materials-Oriented Chemical Engineering, Nanjing Tech University

No.30 Puzhu South Road, Nanjing, 211800, China

Tel: (86) 25-58139942

Email: biotech@njtech.edu.cn

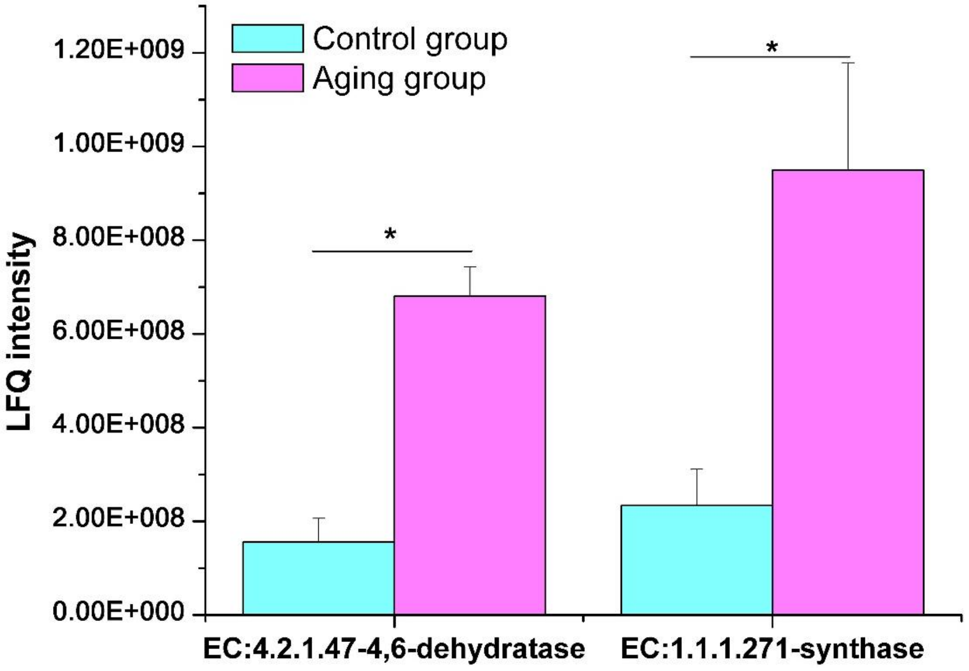

Figure S1 The LFQ intensities of EC:4.2.1.47-4,6-dehydratase and EC:1.1.1.271-synthase (*, $p<0.05$).
